# Molecular and Cellular Mechanisms of Synaptogenesis and Synaptic Refinement During Cerebellar Circuit Formation

**DOI:** 10.3390/ijms27177576

**Published:** 2026-08-24

**Authors:** Farshid Ghiyamihoor, Azam Asemi Rad, Hassan Marzban

**Affiliations:** 1 Department of Human Anatomy and Cell Science, Rady Faculty of Health Sciences, Max Rady College of Medicine, University of Manitoba, Winnipeg, MB R3E 0J9, Canada; 2 The Children’s Hospital Research Institute of Manitoba (CHRIM), Rady Faculty of Health Sciences, University of Manitoba, Winnipeg, MB R3E 3P4, Canada

**Keywords:** synaptogenesis, synaptic pruning, cerebellar circuitry, CBLN1, GluD2, neurexin, neuroligin

## Abstract

Interactions among molecular recognition systems, neuronal activity, and glial regulation transform early neuronal connectivity into precise functional circuits during brain development. The cerebellum is a powerful model for studying these mechanisms due to its stereotyped and accessible circuitry. Two major excitatory afferent pathways—climbing fibers (CFs), which convey error-related signals to Purkinje cells (PCs), and mossy fibers (MFs), which transmit sensorimotor information via granule cells (GCs) and parallel fibers (PFs)—undergo strengthening, competition, and refinement during postnatal development. Synaptic specificity is established by general and pathway-specific organizers. The neurexin–neuroligin system broadly regulates synapse formation, while the neurexin–CBLN1–GluD2 complex specifies PF–PC synapses and C1qL1–BAI3 signaling stabilizes the dominant CF input during competitive refinement. CF–PC synapse elimination serves as a classic model of activity-dependent competition, where weaker inputs are removed through calcium-dependent mechanisms. In parallel, glial cells regulate synaptic maturation: microglia shape inhibitory environments, and Bergmann glia support glutamate homeostasis, dendritic organization, and synapse stability. PCs integrate CF and PF inputs and provide inhibitory output to the cerebellar nuclei, where convergent excitatory collaterals from CFs and MFs are combined with PC inhibition to generate cerebellar output. Together, these coordinated molecular, cellular, and circuit-level mechanisms establish the synaptic architecture underlying cerebellar computation, motor coordination, and adaptive learning, which are the central focus of this review.

## 1. Introduction

Neural circuits are not born precise. They emerge through phases of synapse overproduction followed by selective stabilization, a developmental strategy that transforms initially redundant connectivity into efficient and reliable networks. The cerebellum provides a particularly powerful model for studying these processes because its circuitry is anatomically well defined, developmentally staged, and functionally dependent on precise synaptic refinement. These mechanisms are essential for coordinated motor control and increasingly recognized as contributors to cognitive and affective function [1,2,3,4].

The cerebellum originates from rhombomere 1 around 30 days post-conception in humans (embryonic day (E) 8 in mice) [5]. Two germinal zones generate the principal neuronal populations of the cerebellar cortex and cerebellar nuclei (CN): the rhombic lip produces glutamatergic neurons, including CN projection neurons, granule cells (GCs), and unipolar brush cells, whereas the ventricular zone generates GABAergic populations, including Purkinje cells (PCs), GABAergic nucleo-olivary neurons, and interneurons [6,7]. In humans, cerebellar neurogenesis features an expanded subventricular zone with basal progenitors that amplify GABAergic production and enable greater foliation, a feature that is absent in rodents [5]. Recent evidence from mouse models suggests that early α-synuclein-positive neurons originate from mesencephalic sources and contribute to CN assembly [8] (Reviewed in [9]).

**Figure 1:**
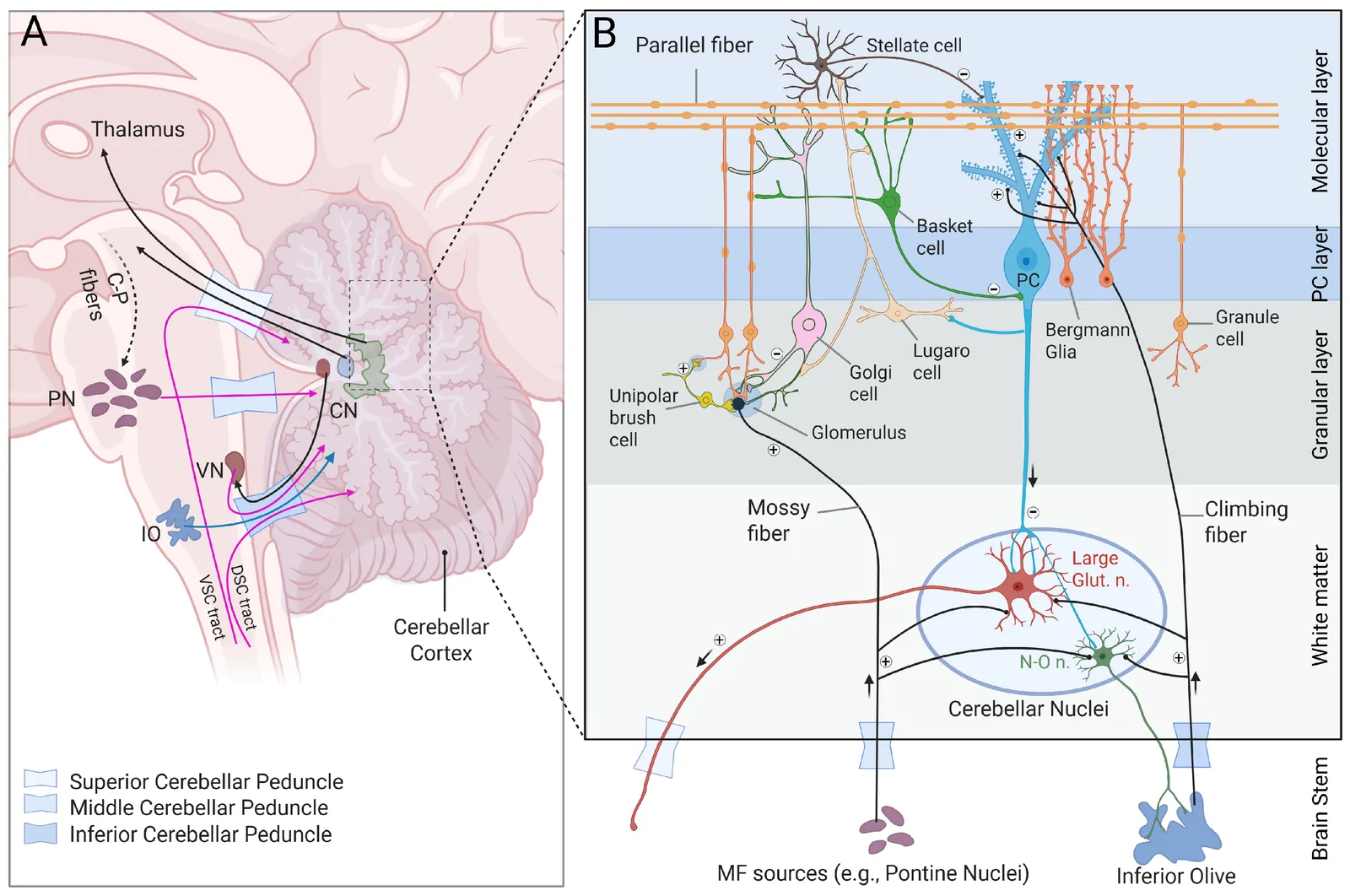
Organization of the mature cerebellar circuit. (**A**) Mossy fibers (MFs) arise from multiple brainstem (e.g., pontine and vestibular) and spinal cord nuclei, whereas climbing fibers (CFs) originate exclusively from the inferior olive (IO). The cerebellar nuclei (CN) constitute the primary output center of the cerebellum. (**B**) MFs innervate granule cells (GCs) and give collateral projections to the CN, whereas CFs form powerful excitatory synapses on Purkinje cells (PCs) and also send collaterals to the CN. Within the cerebellar cortex, MF input to GCs is modulated by Golgi cells and amplified by unipolar brush cells. GC axons, known as parallel fibers (PFs), excite PCs and molecular layer interneurons (basket and stellate cells), which provide feedforward inhibition to PCs. CFs establish powerful excitatory synapses on PC dendrites, where they integrate with PF input to regulate PC activity. PCs, the sole output neurons of the cerebellar cortex, project inhibitory axons primarily to the CN. CN glutamatergic projection neurons constitute the principal output of the cerebellum to the thalamus and brainstem, whereas nucleo-olivary neurons provide inhibitory and excitatory feedback to the IO, completing recurrent cerebellar loops. Figure designed and prepared by F. Ghiyamihoor; Created in BioRender. Marzban, H. (2026) https://biorender.com/fgc6v48.

As these neuronal populations differentiate and migrate, they establish a stereotyped circuit architecture (Figure 1). PCs integrate climbing fiber (CF) and parallel fiber (PF; GC axon) inputs, with the latter receiving excitation from mossy fibers (MFs). The CN integrate excitation from CFs and MFs and inhibition from PCs to generate cerebellar output [10,11]. This circuitry is progressively refined through activity-dependent competition, synapse-organizing molecules, and glial regulation [12,13,14,15].

Although human cerebellar development is prolonged and characterized by expanded progenitor populations and greater foliation, the core architecture and molecular programs governing synapse formation are highly conserved across vertebrates [5,6,16,17,18,19]. This conservation has made genetically tractable mouse models indispensable for defining mechanisms of synaptogenesis, refinement, and circuit assembly [20,21,22]. These findings are increasingly supported by comparative molecular analyses and post-mortem human studies [23,24,25], with recent evidence also revealing abnormal CF–PC synapses in disorders such as essential tremor [26]. Subtle species differences, such as primate-specific PC subtypes with distinct synaptic plasticity profiles [16] and human expansions in early-born subtypes [18], highlight translational limitations. Nonetheless, animal models provide a uniquely tractable system for dissecting causal molecular pathways, enabling real-time manipulations and the study of dynamic processes such as microglial-mediated synaptic pruning [27], which cannot be directly examined in human tissue.

Synaptogenesis in the central nervous system (CNS) begins around E15 in rodents, peaks around postnatal day (P) 10, and is followed by synaptic pruning extending into juvenile stages. In humans, these processes begin prenatally (~18 gestational weeks (GW)) and continue throughout childhood and adolescence [28]. This review first synthesizes the core molecular and cellular mechanisms governing CNS synapse formation and refinement before examining how these principles shape cerebellar circuit assembly. Unless otherwise stated, developmental events described here are based primarily on mouse studies.

## 2. Core Principles of Synapse Formation and Refinement in the CNS

Synaptogenesis is a fundamental step in neural circuit assembly that follows neuronal differentiation, polarization, and axon guidance [29]. Early circuits are characterized by an excess of synaptic contacts and axonal branches that are subsequently refined through activity-dependent and glial-mediated stabilization or elimination [13]. The mechanisms summarized in this section represent general principles derived from multiple CNS circuits and should not be assumed to apply identically to all cerebellar synapses; where cerebellar mechanisms diverge from these canonical models, these differences are specified in Section 3.

### 2.1. Axon Guidance and Target Recognition

Postmitotic neurons initially extend multiple neurites before one is specified as the axon and the remainder adopt dendritic identity [30]. This symmetry breaking is controlled by conserved polarity pathways, including LKB1-SAD, PI3K-PIP3, PAR3-PAR6-aPKC, and Rho-family GTPases, which coordinate cytoskeletal organization and intracellular trafficking toward the nascent axon [31,32]. Axonal maturation culminates in the formation of a dynamic growth cone that interprets extracellular guidance cues to direct target recognition [33,34].

Growth cones navigate to targets through conserved ligand–receptor systems, including Netrin, Semaphorin (Sema), Slit, and Ephrin families, together with morphogen gradients such as WNT, SHH (Sonic hedgehog), and BMP (Bone morphogenetic protein) [33,34]. Intracellularly, these signaling cues regulate actin and microtubule dynamics via signaling cascades involving Rho GTPases and second messengers such as calcium and cyclic nucleotides (cAMP, cGMP) [33,34]. In parallel, cell adhesion molecules (CAMs) provide permissive substrates that support axon extension and stabilize contacts along developing pathways [35,36]. Together, these polarity and guidance mechanisms position axons and dendrites for subsequent synapse formation.

### 2.2. Synapse Formation: Adhesion Molecules/Organizers and Synaptic Scaffold Proteins

**Figure 2:**
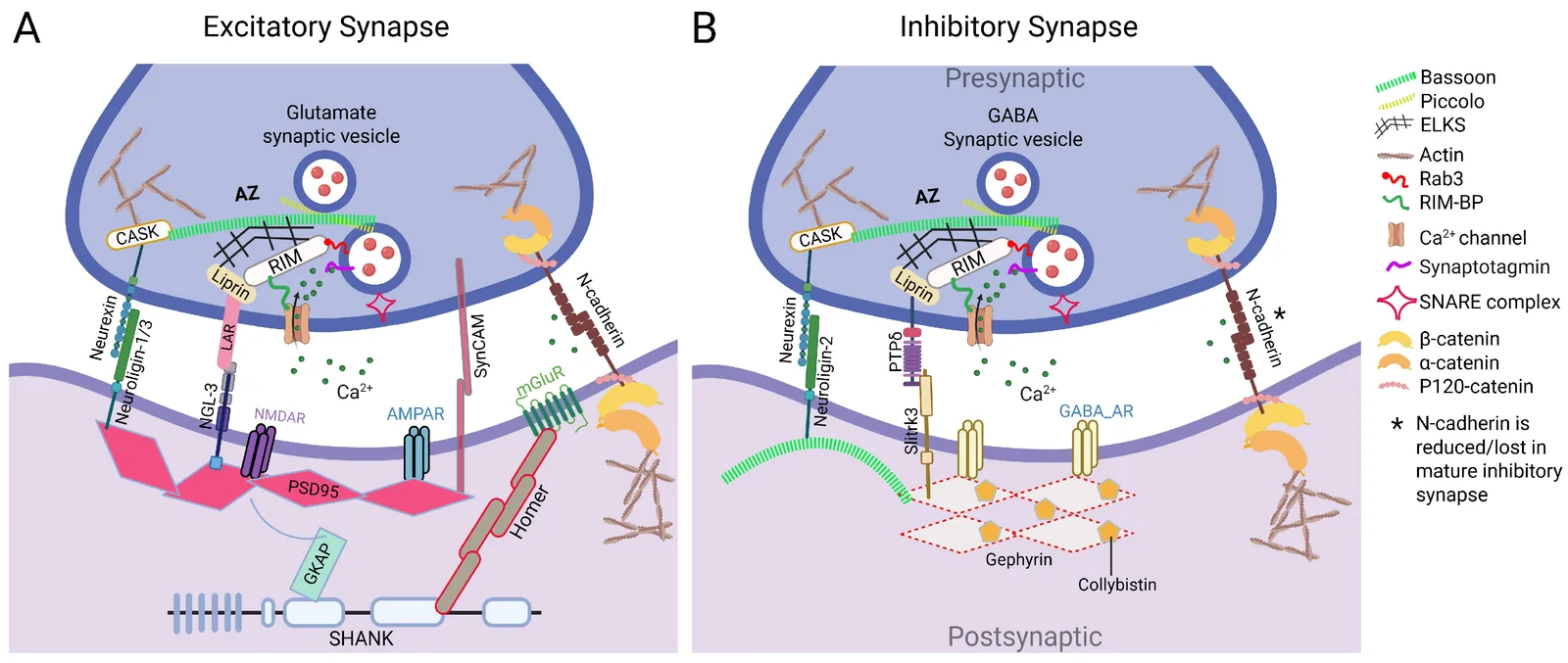
Molecular organization of presynaptic and postsynaptic specializations. (**A**) Excitatory synapse. Trans-synaptic adhesion molecules establish contact between pre- and postsynaptic membranes, promoting synapse specification and alignment. Presynaptic neurexins and LAR receptor protein tyrosine phosphatases (LAR-RPTPs) interact with postsynaptic ligands to organize synaptic contacts. At the presynaptic terminal, glutamate-filled synaptic vesicles are released through a conserved machinery organized at the active zone (AZ). RIM, Munc13, ELKS/CAST, Bassoon, Piccolo, Liprin-α, RIM-binding proteins, and CASK contribute to active-zone assembly, vesicle docking and priming, and positioning of voltage-gated Ca^2+^ channels near release sites. Postsynaptically, AMPA and NMDA receptors are clustered within the postsynaptic density (PSD), where PSD-95 family proteins, GKAP/SAPAP, SHANK, and Homer organize receptors and signaling complexes and link them to the actin cytoskeleton. (**B**) Inhibitory synapse. Distinct trans-synaptic adhesion molecules establish synapse specificity, including neurexin–neuroligin-2 interactions. The presynaptic organization is broadly similar to that of excitatory synapses, but the vesicles contain GABA rather than glutamate and are released from a comparably organized AZ. Postsynaptically, neuroligin-2 and collybistin promote recruitment of gephyrin, which anchors and organizes GABA-A receptor clusters to form the inhibitory postsynaptic scaffold. Figure designed and prepared by F. Ghiyamihoor; Created in BioRender. Marzban, H. (2026) https://biorender.com/021ak1e.

Once axons reach their targets, synaptogenesis begins with initial contacts between axons and immature neuronal processes. Synapse organization is mediated by several families of trans-synaptic adhesion molecules that coordinate pre/postsynaptic differentiation. Major presynaptic organizers include neurexins and leukocyte common antigen-related receptor protein tyrosine phosphatases (LAR-RPTPs, including LAR, PTPσ, and PTPδ), which interact with diverse postsynaptic ligands to establish synaptic identity [37,38]. Prominent postsynaptic organizers include several leucine-rich repeats (LRR)-containing families such as LRRTMs and related adhesion systems, as well as immunoglobulin superfamily members that support synaptic partner recognition and stabilization [39,40] (Figure 2).

Neurexins bind postsynaptic partners such as neuroligins, cerebellins (CBLNs)–GluD receptor complexes, LRRTMs/FLRTs, and NGLs, forming trans-synaptic bridges that coordinate presynaptic vesicle release machinery with postsynaptic scaffolds including PSD-95 at excitatory synapses and gephyrin at inhibitory synapses, thereby contributing to synapse specification and maturation [37,38]. LRRTM-mediated signaling primarily promotes excitatory synapse formation by facilitating AMPA (α-amino-3-hydroxy-5-methyl-4-isoxazole propionic acid) receptor recruitment and postsynaptic density maturation [41,42,43]. In parallel, LAR-RPTPs interact with multiple postsynaptic ligands, including tropomyosin receptor kinase (Trk) C, SALMs, NGL-3, and Slitrks, thereby contributing to synapse specification and maintenance of excitatory–inhibitory balance [39,40,44,45] (Figure 2). Importantly, human and animal genetic studies have linked several synaptic organizers, including neurexins [46,47], neuroligins [48,49], and PTPδ (gene: *PTPRD*) [50,51,52], to neurodevelopmental disorders such as autism spectrum disorder (ASD), intellectual disability, attention-deficit/hyperactivity disorder (ADHD), and schizophrenia, underscoring the importance of precise trans-synaptic signaling during circuit assembly.

Additional adhesion systems, including cadherins, protocadherins, and SynCAMs, link trans-synaptic recognition to cytoskeletal organization and synapse stabilization [39,40,53,54]. Through combinatorial expression and homophilic or isoform-selective interactions, cadherins and protocadherins provide a molecular “barcode” that helps match appropriate pre- and postsynaptic partners [54]. At excitatory synapses, N-cadherin forms a transsynaptic adhesion complex with β-catenin that helps stabilize synapses, regulate postsynaptic organization, and influence AMPA receptor abundance [55,56]. At inhibitory synapses, cadherin/catenin involvement is more heterogeneous, and β-catenin may be retained through other cadherins or adhesion complexes rather than N-cadherin specifically [53,55,56,57].

Early during presynaptic differentiation, synaptic vesicle proteins such as synaptophysin, synaptotagmin, and synapsins accumulate at nascent presynaptic sites, reflecting the initial clustering of synaptic vesicles [10,58,59,60]. This is accompanied by the assembly of presynaptic cytomatrix scaffold proteins at the active zone, including Liprin-α, Rab3-interacting molecule (RIM), Munc13, ELKS/CAST, Bassoon, Piccolo, RIM-binding proteins, and Calcium/Calmodulin-dependent serine protein kinase (CASK), which coordinate vesicle docking and priming, promote SNARE (soluble N-ethylmaleimide-sensitive factor attachment protein receptor)-complex assembly, and position Ca^2+^ channels near release sites [39] (Figure 2A,B).

At excitatory synapses, postsynaptic nano-architecture is organized by an interconnected scaffold centered on MAGUKs such as PSD-95, PSD-93, SAP102, and SAP97, which cluster AMPA- and NMDA (N-methyl-D-aspartate)-type glutamate receptors and couple them to signaling and adhesion complexes. PSD-MAGUKs connect through GKAP/SAPAP to SHANK family scaffolds, forming a PSD-95–GKAP–SHANK axis that extends from the membrane-proximal receptor layer into the actin-rich spine core [39,61,62]. SHANK proteins, in turn, bind Homer tetramers, which link receptor complexes to type-1 metabotropic glutamate receptors (mGluR1), IP3 receptors, and other effectors, thereby integrating ionotropic and metabotropic signaling and coordinating structural remodeling of dendritic spines during synapse maturation (Figure 2A) [61,62,63]. Importantly, disruption of postsynaptic scaffold proteins has been implicated in human neurodevelopmental disorders, with SHANK3 strongly associated with ASD [64,65] and PSD-95 implicated in both ASD and schizophrenia [66,67,68].

At inhibitory synapses, postsynaptic scaffold assembly centers on gephyrin, a self-assembling lattice-forming protein that anchors GABA_A and glycine receptors beneath the postsynaptic membrane in alignment with presynaptic release sites [69,70,71]. Gephyrin organization is regulated in part by neuroligin-2 and collybistin, which couple trans-synaptic adhesion to scaffold recruitment and receptor clustering, thereby shaping the size and stability of inhibitory postsynaptic nanodomains [39,69,70,71] (Figure 2B). Together, these interactions determine inhibitory synaptic strength and contribute to the molecular specialization of distinct classes of inhibitory synapses.

Following adhesion and scaffold assembly, nascent synapses undergo functional maturation, transforming structurally assembled proto-synapses into stable and competent signaling units [40]. Presynaptically, maturation enhances the organization of active zones, promotes efficient vesicle release, and strengthens coupling between Ca^2+^ influx and neurotransmitter exocytosis [72]. In many developing CNS circuits, but not necessarily in the cerebellum, postsynaptic recruitment of AMPA receptors converts NMDA receptor-only silent synapses into functional excitatory synapses, while PSD-95–GKAP–SHANK–Homer scaffolds coordinate receptor organization with actin cytoskeleton remodeling to support synaptic plasticity and spine maturation [61,73]. Consistent with this model, PSD-95 knockout mice maintain ~50% of synapses in a prolonged NMDA receptor-only silent state into adulthood [74]. Inhibitory synapses mature through stabilization of GABA_A receptor clusters by neuroligin-2–gephyrin complexes [61,71,75]. Synaptic refinement begins soon after synapse formation, overlapping with maturation as intrinsic assembly mechanisms transition to activity-dependent strengthening or elimination of synaptic connections.

The role of postsynaptic NMDARs in the cerebellum requires particular caution because their expression is strongly dependent on cell type, synapse, developmental stage, and species [76,77,78]. During early development, PCs express functional GluN2D-containing NMDARs [76,77], whereas functional postsynaptic NMDAR currents in mature mice have been primarily identified at CF-PC synapses, where GluN2A- and GluN2B-containing receptors contribute to complex-spike signaling and cerebellar plasticity [78,79,80]. Consequently, PF-PC synapses do not necessarily follow the conventional NMDAR-dependent silent-synapse maturation model described for many forebrain excitatory synapses, and NMDAR signaling at PF-PC synapses may involve presynaptic rather than exclusively postsynaptic receptor populations [81].

### 2.3. Activity-Dependent Synaptic Refinement

In many developing circuits, including cerebellar CF–PC connections, multiple presynaptic axons initially converge onto single postsynaptic targets. These supernumerary inputs subsequently compete in an activity-dependent manner, resulting in stabilization of a subset of synapses, termed “winners,” and elimination of others, termed “losers” [82]. Correlated pre- and postsynaptic activity promotes synaptic strengthening through patterned activity and coordination among neighboring spines, whereas weak, isolated, or asynchronous inputs undergo weakening and eventual elimination via AMPA receptor loss and reduced release probability [83,84]. Recent evidence extends this framework by showing that NMDAR-dependent long-term depression (LTD) can itself initiate synaptic pruning: synapses that undergo LTD are preferentially eliminated unless they are protected by input-specific activity from neighboring synapses, indicating that LTD can function as a developmental decision point linking functional weakening to structural removal. In this view, LTD is not only a mechanism of short-term or long-term plasticity but also a mechanism that can bias developmental circuit refinement by selectively targeting isolated synapses for pruning [85,86]. In burst-dominated circuits such as the developing cerebellum, precise burst timing further governs competition: synchronized bursts stabilize winners, mistimed bursts eliminate losers [85,87].

**Figure 3:**
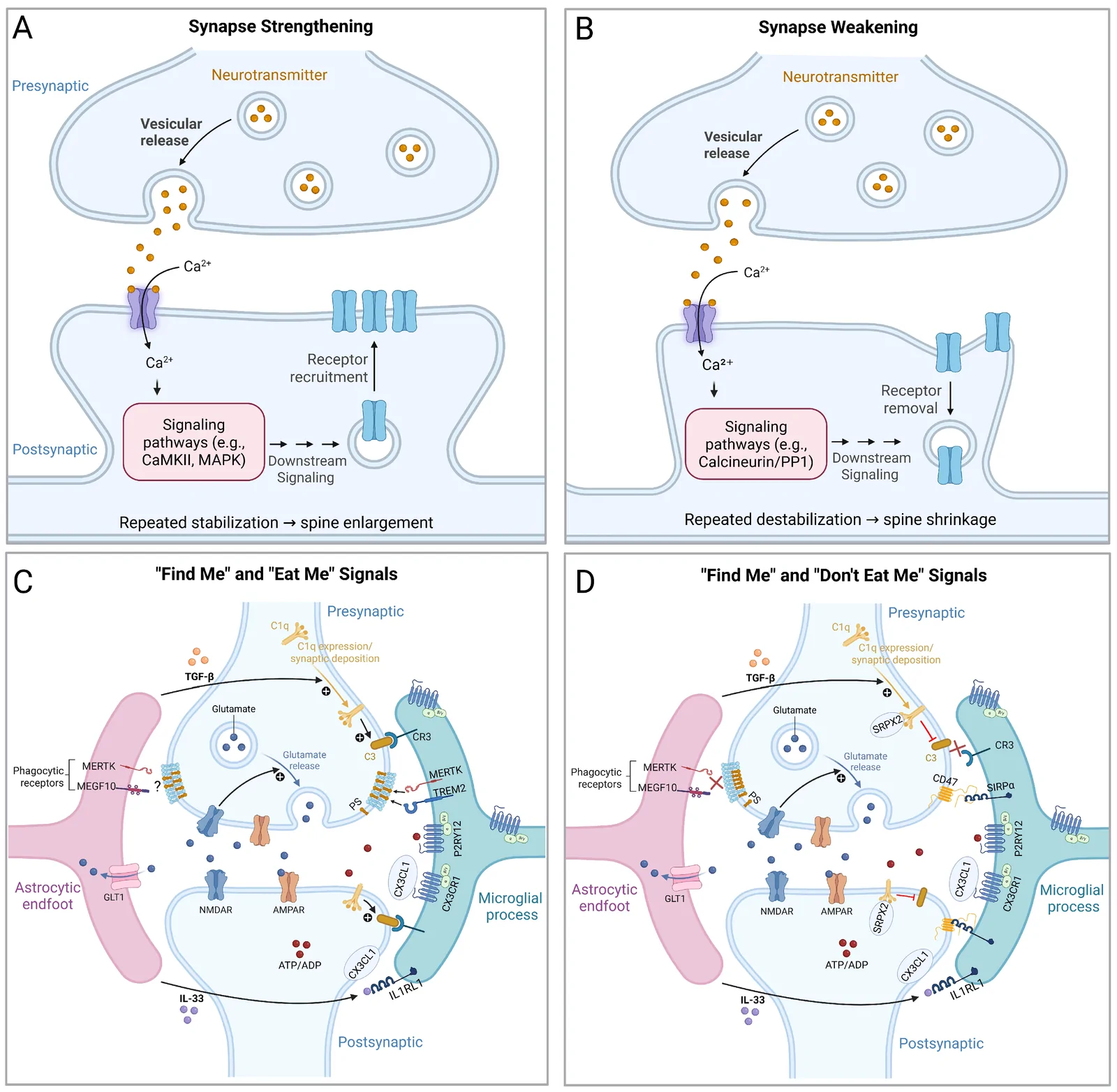
General mechanisms of activity-dependent synaptic refinement and glia-mediated pruning in the CNS. (**A**) Strong, correlated neuronal activity induces transient calcium signaling, promoting receptor stabilization, spine enlargement, and synapse maintenance. (**B**) In contrast, weak or poorly correlated activity leads to receptor destabilization, spine shrinkage, and synapse elimination. Importantly, this canonical kinase/phosphatase framework is not universal: at PF–PC synapses, conjunctive PF and CF activity generates large postsynaptic Ca^2+^ signals and engages mGluR1/PKC-dependent signaling that promotes LTD. Thus, CaMKII and phosphatase signaling in PCs cannot be interpreted using the simple kinase-strengthening/phosphatase-weakening relationship commonly applied to other synapses such as hippocampal glutamatergic synapses. (**C**) Weakened synapses are targeted for elimination through complement- and phosphatidylserine-dependent mechanisms. Microglial surveillance is guided by activity-dependent “find-me” signals, including ATP/ADP and fractalkine (CX3CL1), acting through P2RY12 and CX3CR1. Astrocyte-derived TGF-β induces neuronal C1q expression, triggering C3 deposition and recognition by microglial complement receptor 3 (CR3). Externalized phosphatidylserine provides an additional “eat-me” signal recognized by phagocytic receptors such as TREM2 and MERTK. Astrocytes further regulate pruning through IL-33 signaling and glutamate homeostasis. (**D**) Functional synapses are protected by “don’t-eat-me” signals, including CD47-SIRPα and SRPX2. Figure designed and prepared by F. Ghiyamihoor; Created in BioRender. Marzban, H. (2026) https://biorender.com/4fj37gf.

At the molecular level, activity-dependent calcium signaling serves as a conserved mechanism that guides synaptic refinement. Synaptic activity drives postsynaptic calcium influx. The amplitude, timing, duration, and subcellular localization of intracellular calcium signals are decoded by cell type- and circuit-specific signaling networks. These calcium-dependent pathways regulate the balance between kinase-mediated processes, such as those involving CaMKII, MAPK/ERK, and PKC, and phosphatase-mediated processes, including calcineurin and PP1. Through modulation of AMPAR trafficking, cytoskeletal remodeling, and gene expression, these signaling cascades determine whether synapses are strengthened and stabilized or weakened and destabilized [88]. At the structural level, these changes are reflected in dendritic spine remodeling, where spine enlargement is generally associated with synaptic stabilization and strengthening of connectivity, whereas spine shrinkage often precedes synaptic weakening and elimination (Figure 3A,B) [89]. PF–PC synapses exhibit specialized plasticity rules in which conjunctive PF and CF activity generates large postsynaptic Ca^2+^ signals and engages mGluR1/PKC-dependent signaling that promotes LTD (Section 3.2). CaMKII and phosphatase signaling in PCs therefore cannot be interpreted using the simple kinase-strengthening/phosphatase-weakening relationship commonly applied to canonical hippocampal excitatory synapses.

Neuronal activity also regulates synaptic maintenance through neurotrophic signaling. Brain-derived neurotrophic factor (BDNF) and neurotrophin-3 primarily stabilize postsynaptic developing synapses via TrkB and TrkC receptors, respectively [90,91]. Presynaptic development is likewise shaped by activity-dependent mechanisms. For example, shedding of the ectodomain of signal regulatory protein-α (SIRPα) promotes presynaptic differentiation via calcium/calmodulin-dependent kinases and matrix metalloproteinases [91], while Janus kinase 2 (JAK2) signaling has been linked to the elimination of presynaptic terminals [91,92]. Together, these mechanisms illustrate how correlated activity can drive Hebbian-like (“fire together, wire together”) refinement in many developing CNS circuits [82,91]. However, this framework should not be directly generalized to cerebellar PF–PC plasticity, which follows specialized associative rules involving PF activity, CF-evoked dendritic Ca^2+^ signals, and mGluR1-dependent signaling rather than classical spike-timing-dependent Hebbian mechanisms based on back-propagating action potentials.

### 2.4. Glia-Mediated Synaptic Pruning

Glial cells provide an essential layer of control over synapse maturation and refinement. Microglial pruning is coupled to neuronal activity through “find-me” signals detected by purinergic (P2RY12) and chemokine (CX3CR1) receptors sensing ATP/ADP and fractalkine (CX3CL1), directing microglia to remodel synapses [93,94,95]. Complement proteins C1q and C3 initiate microglial elimination by opsonizing weakened synapses as “eat-me” signals. Microglia express complement receptor 3 (CR3) to recognize and engulf complement-tagged synaptic material [27,93,95,96,97]. Weakened synapses also externalize phosphatidylserine, an “eat-me” signal recognized by microglial receptors TREM2 and MERTK, complementing C1q/C3-mediated tagging [27,93,95] (Figure 3C). Conversely, “don’t-eat-me” signals preserve functional synapses: CD47-SIRPα inhibits microglial engulfment, whereas neuronal SRPX2 limits complement activation by reducing C1q deposition [27,93,95] (Figure 3D). Beyond direct phagocytosis, microglia influence synaptic refinement through trophic support, cytokine signaling, and regulation of local circuit excitability [98].

Astrocytes contribute to synapse formation and refinement through multiple mechanisms. They secrete synaptogenic factors, including thrombospondins and Hevin, that promote synapse formation and maturation [57]. Astrocytes modulate microglial pruning competence by secreting TGF-β and the alarmin cytokine interleukin-33 (IL-33), with TGF-β inducing C1q expression in neurons with weak synapses and IL-33 promoting microglial synapse engulfment [99,100] (Figure 3C). Together, these mechanisms illustrate coordinated astrocyte–microglia regulation of synaptic pruning, whereby astrocyte-derived TGF-β and IL-33 modulate complement signaling and microglial engulfment, linking synaptic state to selective elimination. Whether these signaling interactions operate similarly during cerebellar synaptic (e.g., CF–PC) refinement remains to be established.

Astrocytes can also directly eliminate synapses through MEGF10- and MERTK-mediated phagocytosis [101]. Potential mechanisms may involve phosphatidylserine-mediated or C1q-mediated recognition, as MEGF10 and MERTK are known to participate in phagocytosis of apoptotic cells via these pathways [101,102,103] (Figure 3C); however, whether the same mechanism drives developmental synapse engulfment remains incompletely understood.

### 2.5. Experience-Dependent Plasticity and Transition to Synaptic Stabilization

**Figure 4:**
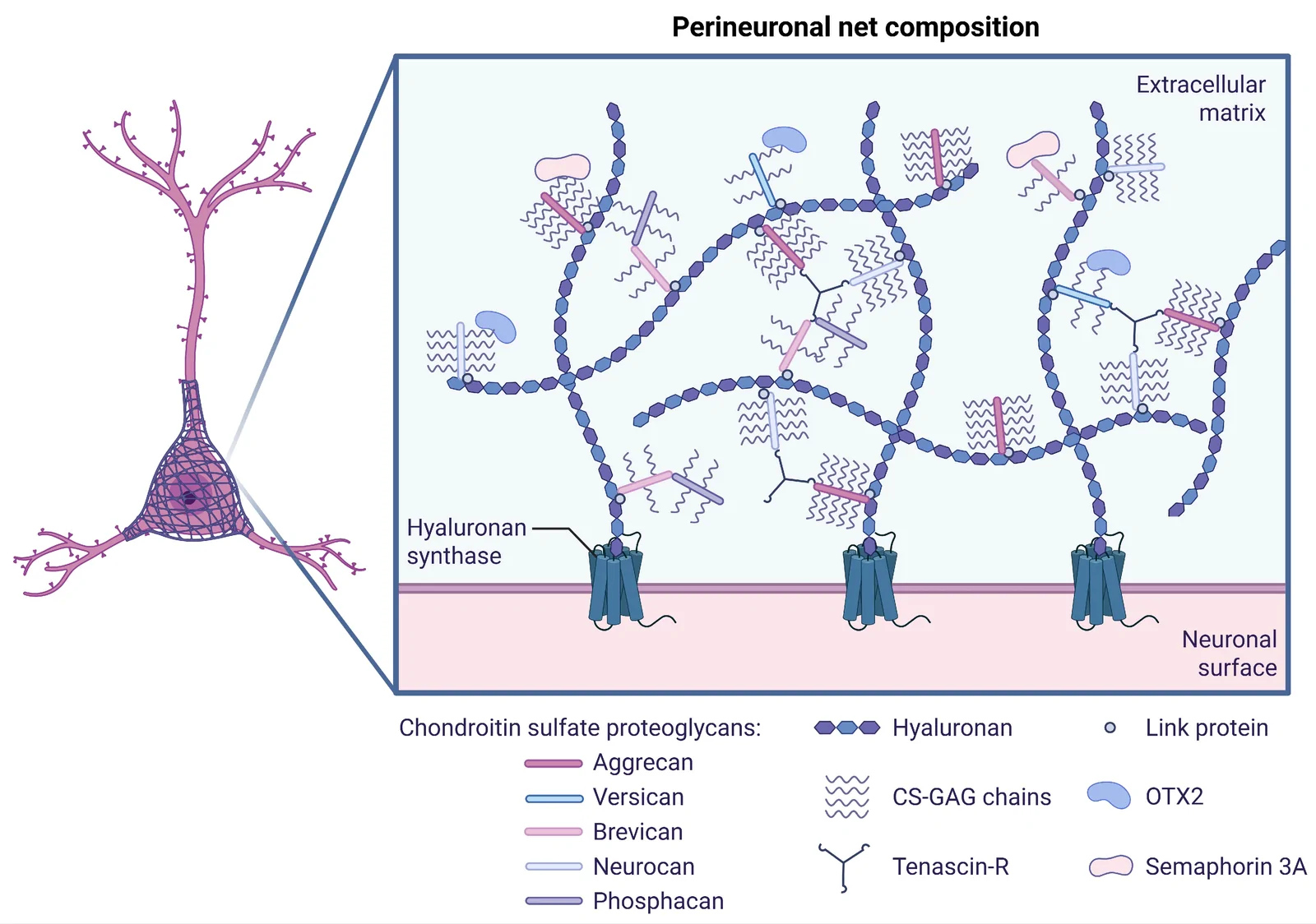
Schematic representation of a perineuronal net (PNN). The left panel shows a neuron ensheathed by a lattice-like PNN surrounding the soma, proximal dendrites, and proximal axon. The right inset magnifies the extracellular matrix, illustrating hyaluronan chains synthesized by membrane-bound hyaluronan synthases and extending into the extracellular space. Chondroitin sulfate proteoglycans (CSPGs), decorated with chondroitin sulfate glycosaminoglycan (CS-GAG) side chains, are anchored to hyaluronan via link proteins, while tenascin-R crosslinks adjacent complexes to form a dense reticular scaffold. Matrix-associated signaling molecules, including Orthodenticle homeobox 2 (OTX2) and semaphorin 3A, are shown bound to the PNN, highlighting its dual role as a structural scaffold and a reservoir for factors that regulate neuronal maturation and synaptic stabilization. Figure designed and prepared by F. Ghiyamihoor; Created in BioRender. Marzban, H. (2026) https://biorender.com/yuhv4oz.

Critical periods are restricted developmental windows during which neural circuits are highly sensitive to environmental stimuli, allowing experience-dependent activity to stabilize appropriate synapses [104,105]. Critical period closure is actively regulated by molecular and cellular programs, including maturation of inhibitory circuits and formation of perineuronal nets (PNNs), which consolidate mature circuit architecture and reduce structural plasticity [106]. PNNs form dense extracellular matrices enriched in hyaluronic acid and chondroitin sulfate proteoglycans that ensheath neuronal somata and proximal dendrites, functioning as a molecular brake on synaptic plasticity and reducing structural plasticity as circuits mature [106,107] (Figure 4). The emergence of PNNs coincides with neuronal and interneuronal maturation, stabilizing circuit architecture by restricting developmental plasticity and promoting critical period closure and long-term circuit stability [108]. After critical-period closure, experience-dependent plasticity persists but is more limited, and homeostatic plasticity plays a larger role in maintaining circuit stability [109,110,111].

## 3. Synapse Formation and Refinement Within the Cerebellar Circuit

Synapse formation and refinement in the cerebellum occur within a highly organized yet developmentally flexible circuit architecture. Rather than refining an immature adult circuit, cerebellar microcircuit development remodels transient neuronal and synaptic connections into mature networks, resembling a metamorphosis [112]. PCs, the principal inhibitory projection neurons of the cerebellar cortex, integrate CF and PF inputs before conveying output to the CN and vestibular nuclei. GCs, the most numerous excitatory neurons in the brain, receive diverse MF inputs, transform them into high-dimensional patterns, and relay them along their PF axons that run orthogonally through PC dendritic arbors (Figure 1B). During development, these neuronal populations establish exuberant synaptic contacts that are subsequently stabilized, remodeled, or eliminated to generate the mature cerebellar microcircuit. Unipolar brush cells amplify MF signals, whereas Golgi, basket, and stellate cells provide inhibitory control of PC output (Figure 1B). Although activity-dependent mechanisms primarily regulate synaptic terminal differentiation, molecules such as Merlin, encoded by *Nf2*, contribute to pre/postsynaptic organization in the cerebellum [113].

### 3.1. MF Afferent Pathway: From Transient MF–PC Contacts to Mature MF–GC–PC Axis

Cerebellar MFs are vesicular glutamate transporter 1/2 (VGLUT1/2)-positive glutamatergic afferents arising from multiple sources, including pontocerebellar, spinocerebellar, vestibulocerebellar, and reticulocerebellar pathways (Figure 1A), which convey diverse sensorimotor information to GC dendrites. The convergence of these inputs enables GCs to generate combinatorial PF output to PCs, expanding cerebellar information processing [114,115].

A key early event in cerebellar development is the entry of trigeminal ganglion pioneer afferents into the cerebellar primordium. These MFs first target the nuclear transitory zone (NTZ; precursor of the CN) at ~E9 and subsequently extend branches toward the nascent PC plate at E10-E11 [116]. This developmental sequence challenges conventional terminology, as the CN-directed branch traditionally termed the collateral is actually the pioneer projection, whereas the later projection to the PC plate represents the true collateral. By innervating early-born NTZ/CN neurons before PCs are generated, these pioneer axons establish the earliest cerebellar connectivity and provide a scaffold for later circuit assembly. These findings support a “nuclei-first” model in which later-born PC axons extend toward the CN by following pre-existing pioneer projections (“backward following”) [114,116,117]. For consistency with the literature, the conventional terminology is retained, referring to the CN-directed branch as the collateral (Section 3.5). Following this pioneer stage, MFs transiently innervate PCs before establishing mature synaptic partnerships with GCs.

#### 3.1.1. Transient MF–PC Synaptic Stage

**Figure 5:**
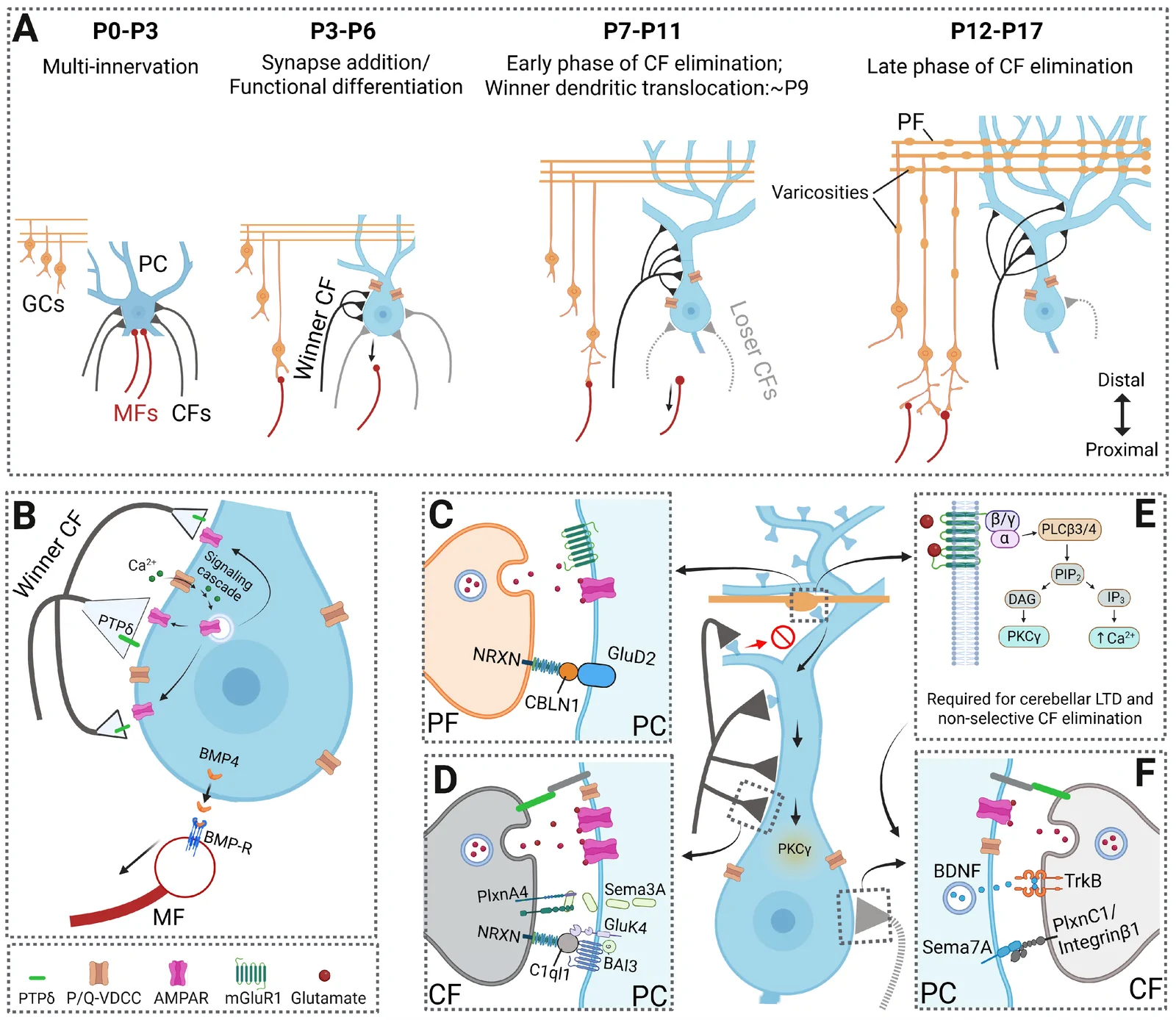
Sequential phases of mossy fiber (MF) and climbing fiber (CF) differentiation and refinement. (**A**) MFs transiently innervate Purkinje cell (PC) somata before shifting to granule cells (GCs), establishing the mature MF-GC synapse. In parallel, PCs are initially innervated by multiple CFs. Between P3 and P6, activity-dependent functional differentiation establishes a dominant winner CF. During the early elimination phase, driven by homosynaptic competition, the winner CF translocates from the soma to proximal dendrites by ~P9, whereas weaker CFs begin to regress. During the late elimination phase, redundant CF inputs are eliminated as parallel fiber (PF) synapses expand onto distal dendrites, establishing the canonical predominantly single-CF-per-PC architecture in rodents. (**B**) Activity-dependent P/Q-VDCC-mediated calcium signaling promotes winner CF differentiation. In parallel, retrograde BMP4 signaling drives withdrawal of transient MF-PC synapses and promotes GC innervation. (**C**) PF-PC synapse formation depends on the neurexin (NRXN)-CBLN1-GluD2 tripartite complex, which stabilizes PF synapses on distal PC dendrites and restricts CF territory to proximal dendrites. (**D**) Anterograde C1qL1-BAI3 and retrograde Sema3A-PlxnA4 signaling contribute to selection and stabilization of the dominant CF. (**E**,**F**) PF activity activates the mGluR1-Gαq-PLCβ3/4-PKCγ signaling cascade (arrows within the PC, the pathway illustrated in (**E**)), promoting elimination of weaker CF inputs through retrograde BDNF-TrkB and Sema7A-PlxnC1/Integrin β1 signaling (shown in (**F**)). Figure designed and prepared by F. Ghiyamihoor; Created in BioRender. Marzban, H. (2026) https://biorender.com/6r0qzn0.

During the early postnatal period in mice, MFs form transient synapses onto PC somata and dendrites before shifting to GC dendrites mostly by the second postnatal week (Figure 5A), while collaterals persist onto Golgi cells and CN neurons [118,119,120] (Figure 1B). These early transient contacts may contribute to the spatial organization of cerebellar afferent inputs.

As cerebellar parasagittal stripes emerge, they provide a spatial framework for patterned MF connectivity [119,121]. Spinocerebellar MFs transiently align with emerging PC parasagittal zones through Eph-ephrin signaling. PCs express ephrin-A2 and ephrin-A5 in complementary parasagittal stripes, whereas spinocerebellar MFs express cognate Eph receptors (e.g., EphA4), enabling contact-dependent matching that stabilizes appropriate projections while repelling mismatched ones. In ephrin-A2/A5 double-knockout mice, PC zones remain intact, but spinocerebellar MFs invade ectopic granular territories, demonstrating that ephrin signaling refines MF topography independently of PC development [119,122]. These findings suggest that PC zones provide the topographic template for spinocerebellar MF organization [122]. Whether the same Eph-ephrin mechanism guides other MF populations remains unknown.

#### 3.1.2. Elimination of MF–PC Synapses and Formation of MF–GC Synapses

In parallel with transient MF-PC interactions, GCs proliferate in the external granular layer and migrate radially along Bergmann glia into the internal granular layer. While migrating inward, GCs progressively replace PCs as the primary postsynaptic targets of MFs and leave their axons anchored in the molecular layer that bifurcate to form the characteristic T-shaped PFs (Figure 5A). Although the molecular mechanisms underlying this transition have been characterized primarily in mice, human developmental studies provide limited temporal context, and direct evidence for human MF–PC synapse elimination remains limited.

PC-derived BMP4 contributes to the elimination of transient MF-PC contacts by acting as a retrograde repulsive cue for pontocerebellar MF terminals [118] (Figure 5B). MFs express BMP receptors, and BMP4 expression in PCs increases between P7 and P14; PC-specific BMP4 loss results in increased MF–PC contacts at P0 that persist abnormally through P14 [118]. This process continues until ~P20 in mice and about 2 years of age in humans, coinciding with completion of GC migration and attainment of adult-like GC layer density [123].

**Figure 6:**
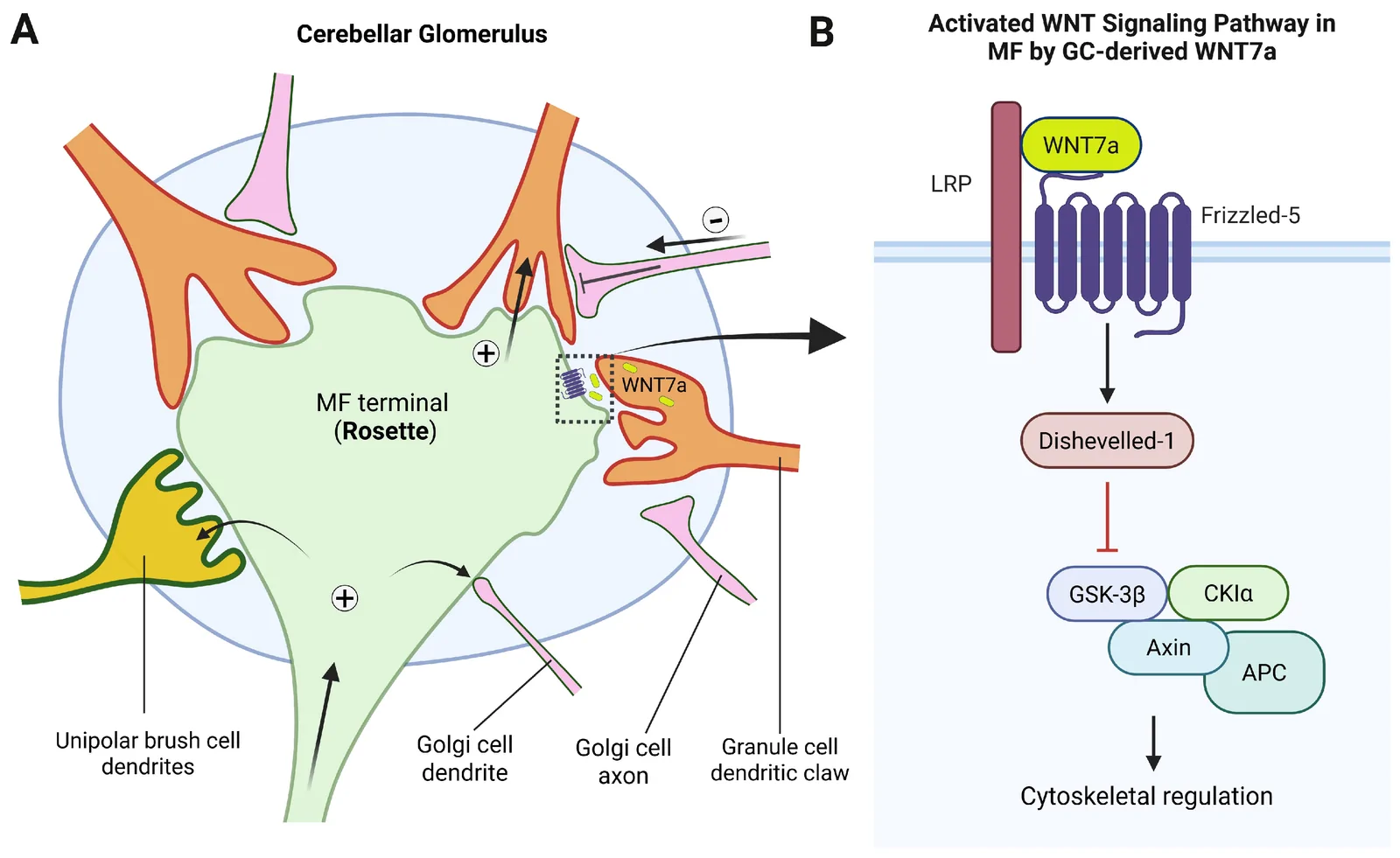
Organization of the cerebellar glomerulus and granule cell (GC)-derived WNT7a signaling regulating mossy fiber (MF) terminal maturation. (**A**) The cerebellar glomerulus is a specialized synaptic complex within the GC layer in which MF terminals enlarge into rosettes in response to GC contact (e.g., WNT7a signaling), forming the mature excitatory MF-GC synaptic unit. Golgi cell dendrites receive excitatory input from the MF, whereas Golgi cell axons provide inhibitory input onto GC dendrites, establishing local inhibitory circuits that regulate GC excitability and signal transmission. (**B**) GC-derived WNT7a acts as a retrograde signal that promotes MF terminal maturation through regulation of cytoskeletal organization. Figure designed and prepared by F. Ghiyamihoor; Created in BioRender. Marzban, H. (2026) https://biorender.com/jbzvgts.

In addition, GCs may recognize their target MFs [124], and their contact triggers MF presynaptic differentiation, promoting their enlargement into rosettes that, together with GC dendritic claws, form cerebellar glomeruli, which first become evident during the first postnatal week in mice and between 26 and 34 GW in humans [59,125] (Figure 1B and Figure 6A). A well-characterized mechanism is GC-derived WNT7a, which acts as a retrograde signal during the second and third postnatal weeks to promote MF terminal maturation. It signals through the Frizzled-5 (FZD5) receptor on MF axon terminals and activates Dishevelled-1 (DVL1) to inhibit Gsk-3β and promote cytoskeletal rearrangements that support rosette growth and assembly of presynaptic components, including synapsin I (Figure 6B) [126,127,128,129]. Accordingly, *Wnt7a*-null mice exhibit delayed synapsin I clustering and immature MF terminals, defects that may be partially compensated by the related ligand WNT7b [130,131].

The developmental WNT7a–FZD5–DVL1 pathway described above should be distinguished from disease-associated alterations in canonical WNT/β-catenin signaling. Aberrant activation of this pathway is a defining feature of the WNT molecular subgroup of medulloblastoma and is frequently associated with activating CTNNB1 mutations and nuclear β-catenin accumulation [132,133]. Whether disruption of the specific GC-derived WNT7a–FZD5–DVL1 pathway contributes to MF–GC abnormalities in medulloblastoma or other cerebellar disorders remains unknown.

FGF22 is another GC-derived retrograde organizer that binds FGFR2 on MF terminals to promote synaptic vesicle clustering and presynaptic differentiation. In vivo, infusion of neutralizing antibodies against FGF22 or *Fgfr2* knockout impairs MF presynaptic differentiation, establishing FGF22–FGFR2 signaling as a key regulator of MF maturation [134]. In addition to target-derived synaptogenic cues, members of the Contactin (Cntn) family, a subgroup of immunoglobulin superfamily CAMs, are sequentially expressed during GC development and contribute to cerebellar circuit assembly by regulating axon guidance, fasciculation, and synaptogenesis in the MF–GC pathway [135]. In particular, Cntn-1 expressed by Golgi cells may help establish the local environment supporting MF-GC synaptogenesis [135].

Developmental timing also contributes to MF-GC specificity. Using PF location-dependent labeling in mice, it has been shown that GCs preferentially synapse with MF populations generated during matching developmental windows, revealing that MF-GC connectivity is temporally biased [125]. Accordingly, earlier-born pontine MFs preferentially innervate middle-layer GCs, whereas later-born dorsal column MFs target superficial-layer GCs, establishing layer-specific glomerular organization for patterned granule expansion [125].

#### 3.1.3. PF–PC Synapse Formation

The horizontally oriented T-shaped PFs in the molecular layer form glutamatergic synapses onto PC distal dendritic spines, establishing the primary site of convergence for GC inputs (Figure 5A,C) [136]. PF–PC synapse formation depends on trans-synaptic adhesion complexes that bridge presynaptic neurexins on PF varicosities with postsynaptic GluD2 (GluRδ2; *Grid2* gene) receptors on PC distal dendritic spines, mediated by GC-secreted CBLN1 [136,137,138]. The neurexin–CBLN1–GluD2 triad not only drives postsynaptic differentiation by recruiting PSD-95 and AMPA receptors while ensuring synaptic specificity and stability during the massive expansion of PF inputs (~100,000 synapses per PC in mice) [12,138]. The contribution of postsynaptic NMDARs to cerebellar synapse maturation requires particular caution because their expression is strongly cell type-, synapse-, species-, and age-dependent. Disruption of this complex, as seen in *Cbln1* or *Grid2* knockout models, leads to profound spine loss, synaptogenesis failure, and cerebellar ataxia [138,139,140]. Additionally, SynCAM1 (gene *Cadm1*) is co-expressed with VGLUT1 in PF-PC synapses, suggesting a role in their formation [141,142]. *Cadm1* knockout mice show a reduced number of PF-PC synapses, cerebellar hypoplasia, and ASD-related symptoms [141,142]. Furthermore, GC-expressed Cntn-6 (NB-3) regulates PF–PC synapse maturation by promoting PF branching and terminal differentiation during peak synaptogenesis (P10–P21). NB-3 deficiency reduces VGLUT1 and mGluR1α puncta density between P5 and P10 [135,143].

Microglia-mediated clearance of excess GCs is a key refinement mechanism in the cerebellar granular layer and is inversely regulated by IL-4 [144]. Elevated IL-4 impairs this physiological pruning, resulting in excess GCs, increased excitatory drive onto PCs, circuit hyperactivity, and impulsive ADHD-like behaviors [144].

#### 3.1.4. Unipolar Brush Cells: Amplification of MF Signal

During the peak of GC radial migration and MF–GC synapse formation, unipolar brush cells (glutamatergic interneurons) enter the internal granule layer by P10 [7] to integrate into maturing glomeruli concurrently with MF–GC synapse refinement (Figure 1B and Figure 6A). Unipolar brush cells are distinguished by a single dendrite ending in a brush-like tuft that forms a giant glutamatergic synapse with a single MF rosette. Postsynaptic AMPA, NMDA, and mGluR1α/2 receptors amplify and prolong MF bursts into temporally diverse signals that are relayed via branched axons to the claws of multiple GCs. Unipolar brush cell axons constitute a substantial fraction of glomeruli cores, particularly in vestibulocerebellar regions, amplifying single MF inputs through feedforward circuits [145]. Molecular cues guiding unipolar brush cells–MF synapse assembly require further investigation.

#### 3.1.5. Golgi Cells: Feedforward and Feedback Inhibition of GCs

Golgi cells, the principal GABAergic interneurons of the granule layer, gate MF input through feedforward inhibition (MF → Golgi → GC) mediated by basal dendrites and through feedback inhibition (PF → Golgi → GC) driven by apical PF inputs (Figure 1B and Figure 6A) [146,147]. Basal dendrites sample multiple MF rosettes, while apical dendrites integrate PFs and CFs [148,149]. In mice, Golgi–GC inhibitory synapses form by P4, concurrent with the peak of MF–Golgi glutamatergic synaptogenesis (P5–P10), which is stabilized through activity-dependent NMDA/AMPA signaling [150]. Mature Golgi cells (post-P16) exhibit biphasic responses to glutamate spillover from PFs/CFs onto apical dendrites: initial excitation via AMPA-mediated depolarization, followed by mGluR2/GIRK-mediated hyperpolarizing pauses that prevent GC overexcitation while promoting sparse, decorrelated PF output for pattern separation [151,152,153].

### 3.2. CF Afferent Pathway Targeting PCs

Among cerebellar afferent pathways, the CF–PC connection provides the best-characterized model of synapse formation, strengthening, and activity-dependent refinement.

#### 3.2.1. CF–PC Synapse Formation

Cerebellar CFs are VGlUT2–positive glutamatergic inputs from inferior olivary neurons that synapse onto PCs, with collaterals onto CN neurons (Section 3.5). During ~P0–P7, each PC is contacted by several CFs that form relatively weak and sparse synaptic contacts primarily on the PC soma. The presynaptic LAR-RPTP family member PTPδ localizes to CF terminals from P0 and is required for normal CF-PC synapse formation and maturation in the anterior cerebellum (Figure 5B) [154]. *Ptprd* knockout mice exhibit reduced CF innervation per PC and impaired CF dendritic translocation. These mice also show reduced VGlUT2 puncta size and overlap with the active zone marker RIM1/2, indicating smaller presynaptic terminals and impaired active zone assembly because PTPδ recruits liprin-α to assemble active zone proteins, including RIM1/2 and CASK [154]. This confirms that PTPδ establishes multi-CF contacts and promotes active zone maturation [154]. Between P3 and P7, individual CFs preferentially add numerous synapses onto a subset of PC targets via a positive-feedback addition mechanism modeled as “rich-get-richer”, without evidence of synapse loss from other targets or differences in active zone size between strong and weak inputs [155]. This quantitative synapse accumulation represents the primary form of early developmental plasticity in this circuit [155] (Figure 5A,B). These processes establish the structural foundation for subsequent refinement.

#### 3.2.2. CF–PC Synaptic Pruning

At P3, each PC receives multiple motile CF inputs on its soma that rapidly stabilize on dendrites. Redundant CFs are refined to predominantly single-input innervation through three phases: activity-dependent functional differentiation establishing a winner CF, an early phase of CF elimination independent of PF–PC synapse formation, and a late phase of CF elimination requiring normal PF–PC development [13] (Figure 5A,C).

Functional differentiation (P3–P6): In parallel with quantitative synapse accumulation [155], multiple CFs innervating each PC undergo functional differentiation without numerical reduction. One winner CF emerges as stronger through greater activity-dependent calcium influx via postsynaptic P/Q-type (Cav2.1) voltage-dependent calcium channels (P/Q-VDCCs), preferentially allocating synaptic resources, including AMPA receptor insertion, to the most active input while others weaken [156,157,158] (Figure 5A,B). This phase establishes strength disparities through homosynaptic competition. PC-specific *Cacna1a* knockout mice lacking P/Q-VDCCs exhibit persistent strengthening and dendritic translocation of multiple CFs and impaired elimination by P12 [13]. In humans, pathogenic *CACNA1A* variants cause several neurological disorders including familial hemiplegic migraine, episodic ataxia type 2, and spinocerebellar ataxia type 6 (SCA6) [159,160,161,162]. *CACNA1A*-associated familial hemiplegic migraine is frequently accompanied by cerebellar signs such as ataxia, nystagmus, and cerebellar atrophy [159]. Selecting the winner CF during the functional differentiation phase is independent of CF–PC synaptic transmission, although it is essential for its subsequent dendritic expansion and loser elimination [163].

Early-phase CF elimination (P7–P11): This phase begins as CF inputs per PC decline, driven by P/Q-VDCC-mediated Ca^2+^ signaling. This activity-dependent bias is accompanied by structural remodeling: the strongest CF translocates from the soma to the proximal dendrites by ~P9, whereas weaker somatic CFs regress, establishing a competitive relationship with PF inputs [164,165] (Figure 5A). This process is potentially supported by IGF-1 (insulin-like growth factor 1) and TrkB–BDNF signaling. IGF-1 promotes CF synapse strengthening and dendritic translocation, whereas reduced TrkB signaling—characterized by increased expression of the truncated TrkB isoform and decreased levels of full-length and phosphorylated TrkB—facilitates the elimination of weaker CFs [13,166]. A stabilization mechanism spanning the early and late elimination phases involves Sema3A–PlxnA4 signaling. PC-secreted Sema3A binds PlxnA4 on CFs and stabilizes CF–PC synapses, as PC-specific Sema3a knockdown or CF-specific Plxna4 knockdown accelerates synapse elimination from P8 to P18 (Figure 5D).

Late-phase CF elimination (P12–P17): This phase completes pruning of perisomatic CF synapses and predominantly depends on PF–PC synapse formation. Two PF–PC synapse-dependent mechanisms drive this process. First, the neurexin–CBLN1–GluD2 tripartite complex stabilizes PF-PC synapses. This stabilization restricts CF innervation to proximal dendrites and soma, indirectly promoting perisomatic CF pruning by limiting expansion space for weaker CF (Figure 5C,D) [13]. Second, PF-driven signals activate mGluR1, triggering a Gαq–PLCβ3/4–PKCγ cascade in PCs that generates LTD of the PF-PC synapse (known as ‘cerebellar LTD’) [167,168,169]. Importantly, at mature PF-PC synapses, cerebellar LTD depends on kinase signaling, including PKC and CaMKII, whereas postsynaptic LTP requires phosphatase activity, including calcineurin/PP1 [170,171,172]. This inverted kinase-phosphatase relationship differs from the canonical model at many forebrain excitatory synapses, although the specific contribution of CaMKII and calcineurin/PP1 signaling to developmental PF-PC synapse elimination remains unclear. During development, the mGluR1-mediated LTD initiates heterosynaptic depression signals, with the downstream molecules BDNF [173] and Sema7A [174] mediating retrograde signaling through their respective receptors, TrkB and PlxnC1/Integrinβ1, to drive CF synapse elimination (Figure 5E,F).

Retrograde BDNF–TrkB signaling functions as an active “punishment” signal during the late phase (post-P16) to enforce final CF elimination [173]. PCs remain multi-innervated by CFs in mice with CBLN1 deletion, mice with PC-specific deletion of GluD2, mGluR1, PLCβ4, PKCγ, BDNF, or Sema7A, or mice with CF-specific TrkB knockdown [13,145,173,175]. NMDA receptors at the MF–GC synapse may also contribute to the final stage of CF elimination during P15–P16 [176], although this mechanism is distinct from the debated expression of postsynaptic NMDARs in PCs.

Recent work has demonstrated that mGluR1-PKCγ signaling is required for functional and structural maturation of “winner” CF, including their selective strengthening, AMPAR accumulation, long-term potentiation, and dendritic territory expansion [177]. Furthermore, mGluR1-PKCγ signaling promotes segregation of CF and PF territories on PC dendrites (P15 onwards) by eliminating competing PF synapses from proximal dendrites, thereby minimizing overlap between these excitatory inputs [178].

Late-phase CF elimination requires further stabilization of the “winner” CF through several retrograde mechanisms. Sema3A–PlxnA4 signaling, introduced above as a mechanism spanning early- and late-phase elimination, continues to support CF stabilization during this period [174]. In addition, progranulin-Sort1 signaling during P11-P16, in which PC-derived progranulin acts retrogradely onto its putative receptor Sort1 on CFs, contributes to “winner” selection [179]. CF-derived C1qL1 (C1q-like 1) binds the PC-expressed BAI3 (brain-specific angiogenesis inhibitor 3), a cell adhesion G-protein–coupled receptor, to promote stabilization of the dominant CF [180,181] (Figure 5D). Interestingly, the PC-expressed GluK4 subunit of kainate-type glutamate receptor binds to C1qL1 to stabilize C1qL1 and BAI3 at synapses [182] (Figure 5D). Activity-dependent accumulation of C1qL1 at CF terminals enables “winner” CF to preferentially activate C1qL1-BAI3 signaling, stabilizing their synapses and, under appropriate activity and expression levels, promoting new synapse formation between CF transverse branches and neighboring PCs, even in the mature cerebellum [183]. In mice lacking CF-specific C1qL1 or PC-specific BAI3, early CF elimination proceeds largely normally; however, the dominant CF fails to stabilize on proximal dendrites, resulting in persistent multiple CF innervation from P14 into adulthood [13,182].

Although these three phases provide a useful framework for CF refinement, their molecular regulation is temporally overlapping rather than strictly stage-restricted. P/Q-VDCC signaling contributes to functional differentiation and subsequent elimination, while TrkB signaling has distinct stage-dependent roles, with reduced TrkB signaling facilitating earlier elimination and BDNF–TrkB retrograde signaling promoting late-phase pruning. Similarly, Sema3A–PlxnA4 signaling spans the early-to-late elimination period (P8–P18), primarily supporting stabilization of the dominant CF. Thus, these pathways are distinguished here by their predominant functional roles rather than by mutually exclusive developmental windows. Likewise, the structural outcome of CF refinement should not be viewed as an invariant one-CF-per-PC endpoint. Although developmental CF elimination produces predominantly mono-innervated PCs in rodents, this canonical organization is not universal. Adult mice with multibranched PC dendritic morphology can retain multiple CF inputs, and recent anatomical analyses suggest that human multibranched PCs may likewise receive distinct CFs onto separate dendritic compartments [184,185].

Collectively, mutant mouse models reveal distinct but overlapping requirements for these pathways during CF refinement. Disruption of P/Q-VDCC or PF-dependent signaling generally impairs CF elimination and results in persistent multiple innervation, whereas Sema3A–PlxnA4 disruption accelerates elimination. In contrast, loss of C1qL1–BAI3 signaling primarily impairs stabilization of the dominant CF. These phenotypes highlight the balance between elimination of weaker inputs and stabilization of the selected CF. Consistent with the functional importance of this balance, increased NRXN1 expression and enrichment at CF-PC synapses in a mouse model carrying an ASD-associated copy-number duplication (*patDp/+*) were associated with impaired PF-PC LTD, ectopic CF synapses on distal PC dendrites, enhanced CF activity, and exaggerated sensory-evoked motor responses [186].

Consistent with the importance of these pathways in experimental models, human variants in genes involved in late-phase CF elimination are linked to multiple cerebellar disorders. Mutations in *GRID2* (GluD2) cause developmental cerebellar ataxia with cerebellar atrophy, intellectual disability, and oculomotor abnormalities [187,188,189,190,191]. Variants in *GRM1* (mGluR1) underlie both autosomal dominant SCA44 [192,193] and autosomal recessive SCAR13, each characterized by cerebellar ataxia and atrophy, with SCAR13 additionally presenting early-onset and neurodevelopmental features [194]. Mutations in *PRKCG* (PKCγ) cause autosomal dominant SCA14, marked by progressive ataxia with myoclonus, dystonia, and spasticity [195,196]. Although SCA14 typically manifests in juvenile to adult stages, PKCγ is essential for both elimination of weaker CF inputs and maturation of the dominant CF(s), suggesting that early developmental defects may contribute to later cerebellar dysfunction. A homozygous intragenic duplication in *ADGRB3* (BAI3) has been associated with intellectual disability, vermis-predominant cerebellar atrophy, ataxia, and behavioral abnormalities [197].

#### 3.2.3. Glial Contribution to CF Pruning

While microglial pruning often involves direct engulfment of synaptic material, cerebellar circuits rely more heavily on indirect regulation of neuronal excitability and inhibitory maturation to drive CF competition and elimination. During development, microglia facilitate late-phase CF elimination by promoting maturation of GABAergic inhibition onto PCs, thereby regulating PC excitability and strengthening competition among CF inputs [14]. Microglia release trophic and inflammatory signals such as BDNF, TNFα, and IL-1β, which enhance maturation of inhibitory interneuron–PC synapses. Consistent with this indirect role, microglial depletion (e.g., *Csf1r* conditional knockout) disrupts inhibitory transmission and impairs CF pruning [14,198], while CX3CR1 signaling is not strictly required for CF elimination [199]. In parallel, Bergmann glia (specialized astroglial population of the cerebellar cortex that ensheath PCs) regulate CF refinement by maintaining glutamate homeostasis and structural ensheathment of PC dendrites through the transporter GLAST (EAAT1; *Slc1a3* gene). In *Slc1a3* knockout mice, impaired glutamate clearance causes retraction of Bergmann glial processes, weakening of primary CF inputs, abnormal strengthening of normally nonsynaptogenic transverse CF branches, persistent multiple CF innervation, and expansion of PF synapses across the PC dendritic arbor [15]. Together, these findings highlight complementary glial mechanisms in CF refinement: microglia influence CF competition through maturation of inhibitory inputs onto PCs, while Bergmann glia regulate glutamate homeostasis and structural ensheathment that shape CF and PF connectivity.

### 3.3. Basket and Stellate Cells: Inhibition of PCs

PFs provide glutamatergic input to basket and stellate cells—GABAergic interneurons of the molecular layer that establish inhibitory synapses on PCs to modulate spike activity [200] (Figure 1B). Basket cells preferentially target the PC soma and axon initial segment, with terminals from multiple basket cells converging to form the pinceau structure that tightly regulates PC spike initiation [200]. This subcellular specificity is supported by distinct targeting mechanisms, including ankyrinG-dependent localization of the adhesion molecule neurofascin-186 at the axon initial segment, which supports basket cell pinceau formation [201]. Stellate cells primarily innervate PC distal dendrites to modulate integration of PF inputs, guided by CHL1 (Close Homolog of L1) signaling associated with Bergmann glial fibers [202].

In mice, interneuron axons begin contacting PCs during the first postnatal week, when nascent boutons assemble presynaptic release machinery while PCs recruit postsynaptic inhibitory scaffolds dominated by gephyrin, stabilized through neurexin–neuroligin-2 adhesion complexes [203]. At these inhibitory synapses, IL-1 receptor accessory protein-like 1 (IL1RAPL1) localizes postsynaptically and interacts with neuronal calcium sensor-1 (NCS-1) to regulate calcium-dependent synaptic responsiveness and timing of inhibitory network maturation, thereby establishing proper excitation–inhibition balance within cerebellar circuits [204]. During the second postnatal week, GABAergic synapses stabilize as receptor clusters enlarge and transmission strengthens. Their functional maturation continues into the third postnatal week alongside a developmental shift in GABA_A receptor composition, particularly α1-subunit enrichment on PCs, supporting faster inhibitory kinetics required for precise cerebellar timing [203].

Once mature, this circuit supports frequency-selective filtering. Low-frequency PF bursts recruit basket cells, which produce sustained GABAergic inhibition onto the PC soma and engage HCN1 (hyperpolarization-activated cyclic nucleotide-gated channels)-dependent shunting, thereby suppressing low-frequency PC firing before excitation reaches the axon initial segment [205,206]. Stellate cells preferentially process higher-frequency inputs, together generating bandpass tuning that refines cerebellar motor control [207].

### 3.4. Purkinje Cells (PCs): Principal Output Neurons of the Cerebellar Cortex

After integrating excitatory and inhibitory inputs, PCs project to the CN, vestibular nuclei, and nucleus prepositus hypoglossi. Before reaching these targets, PCs extend local axon collaterals that innervate Lugaro cells, a small population of inhibitory interneurons located just beneath the PC layer that contribute to inhibitory microcircuits linking the molecular and granular layers. In mice, PC–Lugaro synapses begin to form between P5 and P10. PC GABAergic collaterals provide fast inhibitory input to Lugaro cells, establishing a local feedback circuit. In turn, Lugaro cells release GABA and glycine onto Golgi, basket, and stellate cells, thereby modulating inhibition in both the granular and molecular layers [208,209,210].

The principal targets of PCs are the CN neurons. PC axons begin extending toward the CN primordium (NTZ) ~E13.5 in mice [211], and establish immature perisomatic and proximal dendritic contacts during the first postnatal week. Sparse calbindin-positive terminals co-localized with synaptophysin are detectable by P1, indicating the arrival of PC afferents. By P4, large GAD (glutamic acid decarboxylase)-positive presynaptic patches surround CN neurons, marking the onset of presynaptic differentiation. By P6, coordinated expression of synaptophysin, synapsin I, and GAD confirms functional maturation of inhibitory presynaptic terminals [212]. Between P8 and P12, these immature terminals refine into discrete synaptic contacts while postsynaptic GABA_A receptors and gephyrin become progressively aligned, resulting in mature inhibitory synapses by P12 and into adulthood [212,213]. Proper myelination of PC axons is required for reliable inhibitory transmission at PC–CN synapses, as myelin deficiency reduces presynaptic active zone density and weakens GABAergic input to CN neurons [214].

In addition to CN neurons, subsets of PCs (mainly floccular PCs) target medial and lateral vestibular nuclei and nucleus prepositus hypoglossi [215,216]. As vestibular nuclei share multiple properties with CN neurons, including high spontaneous firing rates and direct innervation by PCs, they have been proposed (especially the lateral vestibular nucleus (Deiters’ nucleus) [217]) to be considered part of CN and, over evolution, they may translocate into the cerebellum.

### 3.5. CN Efferent Pathway: Integration of CF/MF Excitation and PC Inhibition

The CN develop from the embryonic NTZ and include the medial, interposed, and lateral nuclei, which correspond to the fastigial, interposed, and dentate nuclei in humans, and together form the principal output hub of the cerebellum [9,218]. The CN contain several classes of projection neurons, including large glutamatergic, small GABAergic, and glycinergic neurons [218]. These projection neurons integrate glutamatergic inputs from CF and MF collaterals with convergent GABAergic inputs from PCs before sending output to their target regions (Figure 1B).

#### 3.5.1. Excitatory Inputs to CN Neurons from MF and CF Collaterals

MF collaterals provide convergent excitatory input to CN neurons, complementing PC inhibition and helping shape CN spike output. This excitation is best established for glutamatergic projection neurons, while direct innervation of GABAergic nucleo-olivary neurons is less clearly defined [219]. These synapses are individually weak but numerous, so CN output reflects summation across many MF inputs in the context of PC inhibition. Fast transmission is mediated mainly by AMPA receptors, with NMDA receptor components contributing to synaptic integration and developmental maturation. Unlike transient MF–PC contacts in the cortex, MF–CN synapses are retained and convey extracortical sensory and motor information to CN output neurons [219]. CF collaterals excite both large glutamatergic neurons and GABAergic nucleo-olivary neurons within the CN [220,221]. In mice, these inputs are functionally strong by P12–P14, then weaken and become less convergent during postnatal development, consistent with refinement of olivo-cortico-nuclear circuits and maturation of cerebellar output pathways [221,222]. MF-driven excitation in CN neurons is functionally related to the neuron’s CF receptive field, suggesting aligned sensorimotor organization rather than identical topographic mapping [223].

#### 3.5.2. Inhibitory Inputs to CN Neurons from PCs and Local Interneurons

The major inhibitory input to CN neurons arises from PCs, whose calbindin-positive axons make dense GABAergic synapses onto somata and proximal dendrites. This inhibition is dominated by the high-frequency simple-spike firing of PCs, which exerts tonic and temporally precise control over CN activity, whereas complex spikes, triggered by CF input, provide a distinct low-frequency error signal linked to learning and may transiently modulate PC output to the CN [224,225]. Because CN neurons express active conductances, including T-type (Cav3) calcium channels and the hyperpolarization-activated current (I_h_), sufficiently strong inhibitory input can evoke post-inhibitory rebound firing [226,227,228,229]. PC terminals are specialized for sustained transmission: they use spillover from boutons with multiple release sites and relatively modest depression to sustain high firing rates, allowing thousands of inhibitory events per second onto each CN neuron under physiological conditions [230]. Both large glutamatergic projection neurons and small GABAergic nucleo-olivary neurons receive PC inhibition, but the effect differs by cell type: in projection neurons it gates timing, pause responses, and firing, whereas in nucleo-olivary neurons it regulates inhibitory feedback to the inferior olive and helps tune the olivo-cerebellar loop [231].

In addition to the dominant inhibitory input from PCs, CN projection neurons also receive inhibition from local interneurons with mixed GABAergic and glycinergic phenotypes. These interneurons form synapses enriched in glycine receptors that are molecularly distinct from PC synapses (lacking major GABA_A R α1/γ2 subunits) and generate fast inhibitory postsynaptic currents capable of modulating spike timing and output gain [232]. Although representing a smaller proportion of total inhibitory contacts, interneuron-mediated inhibition may contribute to the synchronization of inhibitory events and the generation of rebound firing in CN neurons, thereby refining the temporal precision of cerebellar output [232].

#### 3.5.3. CN Output Stabilization

During the early postnatal period, CN circuitry undergoes activity-dependent refinement as excitatory CF and MF inputs are coordinated with convergent PC inhibition to sculpt functionally defined neuronal ensembles. As these circuits mature, PNNs emerge preferentially around large glutamatergic projection neurons and contribute to long-term stabilization by restricting excessive synaptic remodeling, thereby supporting the transition from a developmentally plastic network to a stabilized output system [233,234]. A recent human study shows that PNNs are selectively enriched within the CN, where they predominantly ensheath excitatory, parvalbumin-expressing projection neurons, supporting that extracellular matrix specialization plays a key role in stabilizing and consolidating refined cerebellar output circuits [235]. Cerebellar PNN manipulation alters synaptic plasticity and intrinsic excitability in the CN, thereby influencing eyeblink conditioning and cerebellar-dependent learning and memory [236]. Thus, the mature CN reflects not only the formation of excitatory and inhibitory synapses, but also their coordinated refinement and extracellular stabilization into a durable and functionally reliable output network.

CN neurons project to several brain regions. The principal output neurons are large glutamatergic neurons and project primarily to the thalamus and several brainstem regions such as the red nucleus and vestibular nuclei [218,237,238]. Cerebellothalamic projections invade the thalamic nuclei by E17.5–E18.5 [239], where information is then relayed to cortical and hippocampal regions, linking cerebellar output to spatial navigation and cognition [240]. Disruption of CN glutamatergic output is sufficient to impair motor coordination and contributes to dystonic movements [237,241]. A second population comprises small nucleo-olivary neurons that project to the inferior olive, forming a feedback loop within the CF pathway [218]. These are predominantly GABAergic in lateral and interposed nuclei [242,243] but also include a smaller glutamatergic subset in the medial nucleus, which projects to the medial accessory olive [244]. The interposed nucleus also contains GABAergic projection neurons that innervate multiple brainstem targets outside the inferior olive, including the pontine nuclei, suggesting an additional feedback loop within the MF pathway [245]. In addition, glycinergic projection neurons comprise at least two subtypes: one, enriched in the medial nucleus, provides ipsilateral projections to vestibular and reticular nuclei [246]; another, described in the lateral CN, extends axons toward the cerebellar cortex and may target Golgi cells, which express glycine receptors on their basal dendrites [247].

Vestibular nuclei, which receive excitatory input from large glutamatergic CN neurons and inhibitory input from PCs, provide predominantly glycinergic projections to the inferior olive [242]. These inputs complement the GABAergic and glutamatergic nucleo-olivary projections and form an additional inferior olive → PC → vestibular nuclei → inferior olive feedback loop.

## 4. Conclusions and Future Direction

Several organizing principles distinguish cerebellar synaptic development from cortical circuit refinement. Cerebellar circuit assembly involves stereotyped developmental target transitions, exemplified by transient MF–PC innervation followed by establishment of the mature MF–GC–PF pathway. CF–PC development involves activity-dependent competition among initially convergent CFs, leading to selection of a dominant input. Importantly, refinement is coordinated across afferent systems, as PF–PC synapse maturation contributes to elimination of redundant CF inputs and segregation of PF and CF territories along PC dendrites. Thus, cerebellar synaptic development is characterized by target switching, competitive refinement, heterosynaptic interactions, and spatial compartmentalization.

The past two decades have revealed coordinated roles for trans-synaptic organizers, neuronal activity, glial cells, and extracellular signaling in cerebellar synapse formation and refinement. Consistent with this molecular diversity, recent synaptome-scale mapping demonstrates a specialized cerebellar “synaptome”, in which distinct combinations of adhesion molecules, receptors, scaffold proteins, and signaling complexes define the identity and functional properties of different synapse populations [248].

Despite these advances, important questions remain. Most mechanistic insights derive from the CF–PC pathway, whereas synapse formation and refinement within the MF pathway and CN remain comparatively underexplored. At the basic-science level, cell-type-specific manipulation combined with emerging circuit-tracing approaches, such as TRACR (transsynaptic anterograde circuit readout) [249], could define how afferents select and refine cerebellar targets. Transcriptomic and spatial approaches could further identify candidate synaptic organizers and signaling pathways within these less-characterized circuits for subsequent functional validation. At the translational level, although animal models remain indispensable for defining mechanisms, their conservation in the human cerebellum requires systematic evaluation. Comparative transcriptomic and connectomic analyses, together with human-based platforms such as cerebellar organoids and assembloids [250], could identify conserved and species-specific developmental programs and enable functional testing of candidate mechanisms. Integrating these approaches with disease-associated genetic and molecular findings may ultimately clarify how disruption of cerebellar synapse development contributes to neurodevelopmental and cerebellar disorders.

## Data Availability

No new data were created or analyzed in this study. Data sharing is not applicable to this article.

## Abbreviations

AMPA	α-Amino-3-hydroxy-5-methyl-4-isoxazolepropionic acid
BDNF	Brain-derived neurotrophic factor
BMP	Bone morphogenetic protein
CBLN	Cerebellin
CF	Climbing fiber
CN	Cerebellar nuclei
GABA	Gamma-aminobutyric acid
Gαq	G-protein alpha q subunit
GC	Granule cell
GluD	Glutamate receptor delta
MF	Mossy fiber
mGluR	Metabotropic glutamate receptor
NMDA	N-methyl-D-aspartate
PC	Purkinje cell
PF	Parallel fiber
PKC	Protein kinase C
PLCβ	Phospholipase C beta
PNN	Perineuronal net
PSD	Postsynaptic density
Trk	Tropomyosin receptor kinase
VGLUT	Vesicular glutamate transporter
